# Dissecting and redesigning enhancers of photosynthesis genes

**DOI:** 10.1093/plcell/koae121

**Published:** 2024-04-22

**Authors:** Peng Liu

**Affiliations:** Assistant Features Editor, The Plant Cell, American Society of Plant Biologists; Donald Danforth Plant Science Center, Saint Louis 63146, MO, USA

Enhancers are DNA sequence motifs that regulate the expression of neighboring genes in response to developmental and/or environmental cues (Schmitz et al. 2022). As a type of cis-regulatory element, enhancers increase the transcription initiation rate when bound by specific transcription factors (TFs). Despite their importance, work on identifying enhancers and characterizing their roles in coordinating the activity of TFs in plants lags significantly behind research in animals. With notable advances in high-throughput sequencing technologies, this presents an exciting opportunity to delve into the study of cis-elements in plants. In this issue, **Tobias Jores, Jackson Tonnies, and colleagues (**Jores et al. 2024**)** have provided an in-depth understanding of how 3 light-responsive enhancers function, both cooperatively and additively.

**Figure 1.**
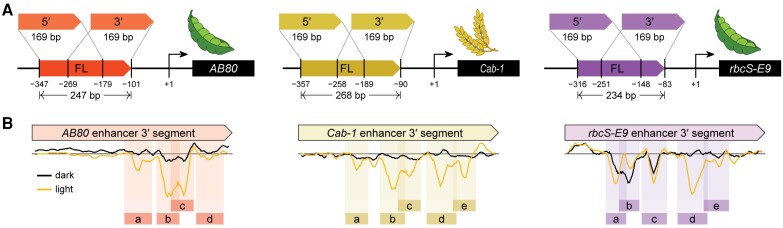
Three enhancers from photosynthesis genes show light-responsive activity. **A)** Enhancers of the pea (*Pisum sativum*) *AB80* and *rbcS-E9* genes and the wheat (*Triticum aestivum*) *Cab-1* gene were cloned to test their enhancer strength. **B)** All possible single-nucleotide substitution, deletion, and insertion variants of 3′ segments of 3 enhancers were subjected to Plant STARR-seq in *N. benthamiana* plants under dark or light conditions. Enhancer strength was normalized to the wild-type variant. Mutation-sensitive regions in the 3′ enhancer segments are indicated by shaded rectangles labeled a–e. Reprinted from Jores et al. (2024), Figures 1A and 3A.

Self-transcribing active regulatory region sequencing (STARR-seq) has emerged as a valuable technique for characterizing enhancers. In previous work, Jores et al. (2020) adapted STARR-seq for use in plants and dissected 3 enhancers associated with photosynthesis genes: the *AB80* and *Cab-1* enhancers that drive the expression of chlorophyI a-b binding proteins, and the *rbcS-E9* enhancer that regulates the expression of a small subunit of Rubisco. They demonstrated that these 3 enhancers exhibit light-responsive activity. Here, the same group of researchers, **Tobias Jores, Jackson Tonnies, and colleagues (**Jores et al. 2024**)**, aimed to interrogate these plant enhancers at nucleotide resolution. They divided each enhancer sequence into 5′ and 3′ fragments (Fig. 1A), conducted saturation mutagenesis on shortened enhancer segments, and measured enhancer strength of all possible single-nucleotide variants under light or dark conditions using the Plant STARR-seq assay. Most mutations had minimal to no effect. However, mutations with a negative effect on enhancer strength tended to cluster together, revealing mutation-sensitive regions (Fig. B). Within these regions, the effect of mutations depended on the surrounding sequence context, suggesting cooperative interactions among individual cis-regulatory elements.

The authors hypothesized that mutation-sensitive regions harbor TF binding sites, and they started with a “motif-scanning” approach to search sequences matching known TF binding motifs. However, this approach failed to capture the majority of binding motifs located within the mutation-sensitive regions. To overcome this, they adopted an alternative approach of integrating saturation mutagenesis data to identify sequence motifs. Enhancer strength data for all single-nucleotide substitution variants were used to create sequence logo plots depicting nucleotide preferences at each position. Remarkably, putative TF binding sites were assigned to nearly all mutation-sensitive regions, highlighting the power of saturation mutagenesis data generated from STARR-seq. The integration approach identified TF binding sequences that frequently exhibited slight deviations from the known TF binding motif, typically differing by 1 or 2 nucleotides. This discrepancy might be the primary reason for the inadequacy of the motif-scanning approach.

Photosynthesis exhibits a circadian rhythm in plants, and *Cab-1* is regulated by the circadian clock. The authors thus investigated whether the *AB80*, *Cab-1*, or *rbcS-E9* enhancers are regulated by the circadian clock. Using Plant STARR-seq, they measured the activity of all single-nucleotide variants of the *AB80*, *Cab-1*, and *rbcS-E9* enhancers in constant light over a 24-hour time course and found that the circadian clock influences the activity of all 3 enhancers. However, they were unable to identify individual mutations in these enhancers that could disrupt their circadian regulation. This finding suggests that the circadian regulation of the enhancers is robust, likely due to the presence of multiple binding sites for TFs controlled by the circadian clock.

The authors further selected 20 short fragments from these 3 enhancers, spanning 1 to 3 mutation-sensitive regions, to investigate how the number, spacing, and order of mutation-sensitive regions affect enhancer strength. Once again, Plant STARR-seq was used, and the results revealed that most individual fragments had minimal to no enhancer activity in both light and dark conditions, consistent with their findings on the cooperativity of mutation-sensitive regions. Additionally, most synthetic enhancers (combinations of 2 or 3 fragments) displayed only weak enhancer activity, indicating that simply combining mutation-sensitive regions is insufficient for cooperative interactions. Further tests revealed that both the spacing between mutation-sensitive regions and the order of enhancer fragments influenced the strength of synthetic enhancers, particularly in light conditions. Fragments spanning 2 mutation-sensitive regions displayed higher activity than the simple combination of individual regions.

Lastly, the authors tested a large number of synthetic enhancers and observed that over one-half of them (1,364) showed activity. From these active synthetic enhancers, they selected 11 and measured their activity in stable Arabidopsis transgenic lines, observing a strong correlation with the enhancer strengths measured by Plant STARR-seq. These findings highlight the feasibility of constructing condition-specific synthetic enhancers using insights derived from Plant STARR-seq data.
